# Discovery of New Inhibitors of eEF2K from Traditional Chinese Medicine Based on In Silico Screening and In Vitro Experimental Validation

**DOI:** 10.3390/molecules27154886

**Published:** 2022-07-30

**Authors:** Qinghua Fu, Xiaomei Liu, Yan Li, Peng Wang, Tian Wu, Haihan Xiao, Yameng Zhao, Qichao Liao, Ziyi Song

**Affiliations:** State Key Laboratory for Conservation and Utilization of Subtropical Agro-Bioresources, College of Animal Science and Technology, Guangxi University, Nanning 530004, China; fqh8630@126.com (Q.F.); lxm01132002@126.com (X.L.); lyyouth0305@126.com (Y.L.); wangpeng131477@126.com (P.W.); wutian980518@126.com (T.W.); xiaohaihan0825@126.com (H.X.); aiyoyo198@126.com (Y.Z.); qc.liao@foxmail.com (Q.L.)

**Keywords:** anticancer, eEF2K inhibitors, pharmacophore screening, molecular docking, traditional Chinese medicine

## Abstract

Eukaryotic elongation factor 2 kinase (eEF2K) is a highly conserved α kinase and is increasingly considered as an attractive therapeutic target for cancer as well as other diseases. However, so far, no selective and potent inhibitors of eEF2K have been identified. In this study, pharmacophore screening, homology modeling, and molecular docking methods were adopted to screen novel inhibitor hits of eEF2K from the traditional Chinese medicine database (TCMD), and then cytotoxicity assay and western blotting were performed to verify the validity of the screen. Resultantly, after two steps of screening, a total of 1077 chemicals were obtained as inhibitor hits for eEF2K from all 23,034 compounds in TCMD. Then, to verify the validity, the top 10 purchasable chemicals were further analyzed. Afterward, Oleuropein and Rhoifolin, two reported antitumor chemicals, were found to have low cytotoxicity but potent inhibitory effects on eEF2K activity. Finally, molecular dynamics simulation, pharmacokinetic and toxicological analyses were conducted to evaluate the property and potential of Oleuropein and Rhoifolin to be drugs. Together, by integrating in silico screening and in vitro biochemical studies, Oleuropein and Rhoifolin were revealed as novel eEF2K inhibitors, which will shed new lights for eEF2K-targeting drug development and anticancer therapy.

## 1. Introduction

Despite advances in diagnosis and treatment, cancer currently remains the leading cause of death in many countries [1]. Therefore, there is an urgent need to explore new therapeutic targets and new molecular targeted drugs. Eukaryotic elongation factor 2 kinase (eEF2K), also known as Ca^2+^/Calmodulin-dependent protein kinase Ⅲ, is an atypical member of the α-kinase family [2,3]. So far, its best-known function is to negatively regulate the extension of polypeptide chain in the process of protein synthesis, through phosphorylation of eukaryotic elongation factor 2 (eEF2) on Thr56 [4,5,6]. Notably, the activity of eEF2K is often found to be enhanced in many types of tumors, and the activated eEF2K in tumor cells is supposed to inhibit protein synthesis, reduces the consumption of nutrients and energy of the cells, thus assisting the tumor cells to resist adverse environments and facilitate their proliferation [7,8]. Consistent with this assumption, a growing number of studies have reported that inhibition of eEF2K expression or activity indeed impairs tumor cells proliferation [9,10]. Therefore, eEF2K is considered as a new potential target for cancer therapy, and the research and development of eEF2K inhibitors are of great clinical significance [11,12,13].

So far, several small-molecule eEF2K inhibitors have been reported. 1-Benzyl-3-cetyl-2-methylimidazolium iodide (NH125) is one of the earliest reported eEF2K inhibitors and it has potent anti-proliferative effects against different tumor cells [14]. However, its contentious effect on eEF2K has not been clearly explained, as eEF2 is phosphorylated rather than dephosphorylated when the kinase activity of eEF2K is inhibited by NH125 [14]. Furthermore, A-484954 is identified as a selective but weak inhibitor of eEF2K. A-484954 decreases eEF2 phosphorylation in cells but has little effect on cancer cell growth [15]. Moreover, several thieno[2,3-b]]pyridine analogs are shown to possess inhibitory activity against eEF2K [16].Additionally, compound **34** is found to be highly effective, with an IC_50_ of 170 nM for eEF2K in vitro, but its mechanism of suppressing eEF2K remains unclear [16].

Recently, computer-aided virtual screening technology is emerging as an excellent tool to advance the development of pharmaceuticals [17]. Compared with the traditional process of new drug discovery, a virtual screen can greatly reduce the scope of artificial ligand activity, thus speeding up the process of drug development and significantly improving the pace of scientific research [17]. In fact, there have been few attempts to perform in silico screening to discover new eEF2K inhibitors. For example, Chen. et al. screened eEF2K inhibitors from FDA approved drugs through virtual screening and found Pemetrexed was a new eEF2K inhibitor [18]. Moreover, Yoshimori., et al. discovered a few novel eEF2K inhibitors using HTS fingerprint which was based on the computer predicted profiling of compound-protein interactions [19]. Although significant progress has been made to seek eEF2K inhibitors in the last 10 years, to date no selective and suitably potent inhibitors of eEF2K have been identified [13].

It is well-known that traditional Chinese medicine (TCM) is a valuable resource for the discovery of molecular-targeted drugs [20]. Extracts prepared from medicinal plants and other natural sources typically contain a variety of molecules with potent biological activities, especially including antitumor activity [20]. For example, astragalus polysaccharides and ginseng polysaccharides, isolated from traditional Chinese herbs Astragalus membranaceus (*A. membranaceus*) and Panax ginseng (*P. ginseng*) respectively, have been proven to have tumor-killing effects [21,22]. However, to date no TCM-derived inhibitors of eEF2K are reported.

To this end, in this study we first screened potential eEF2K inhibitors from traditional Chinese medicine database (TCMD, Version 2009) by integrating pharmacophore screening and molecular docking in Discovery Studio (DS) 2020, and then we picked two purchasable candidates, Oleuropein and Rhoifolin, for further validating the reliability of the screen via in vitro biochemical assays and in silico predictions. The results indicate that our strategy for potential eEF2K inhibitor discovery is effective and reliable. Thus, the finding in this study will contribute to new eEF2K inhibitor discovery, and also give insights into new anticancer drug development.

## 2. Results

### 2.1. Pharmacophore Model Generation and Validation

**Figure 1:**
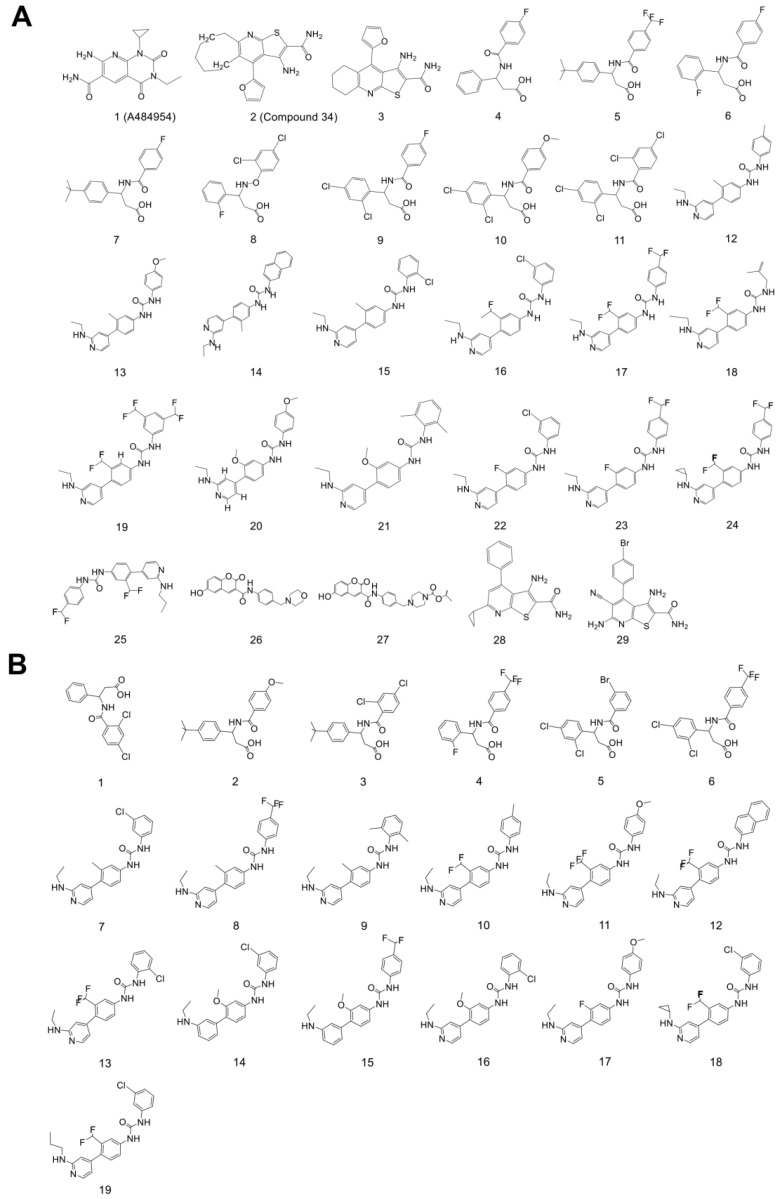
The structures of eEF2K-related chemicals. (**A**) Molecular structures of the training set. (**B**) Molecular structures of the test set, in which **2**, **3**, **12**, **13**, **16**, and **18** are inactive compounds.

To obtain ligand-based pharmacophore models of eEF2K inhibitors, 29 reported eEF2K inhibitors [23,24,25,26] were utilized as the training set (Figure 1A), and then the HipHop tool in DS 2020 was adopted to create the pharmacophore model based on the training set. In the end, a total of 10 pharmacophore models were generated (Supplementary Materials, Figure S1).

Then, to evaluate the reliability of the pharmacophore model, we firstly established a test set of compounds according to the literature [25,26], which includes 13 active chemicals and 6 inactive chemicals (Figure 1B). Subsequently, Ligand Profiler analysis revealed that 08 of pharmacophore model had the highest score (92.31% for HRA) in distinguishing the active chemicals from the inactive ones (Table 1), suggesting pharmacophore model 08 has the best predictive power than the other pharmacophores. Therefore, pharmacophore model 08 was selected for the following analysis.

### 2.2. Virtual Screening Based on Pharmacophore Model

**Figure 2:**
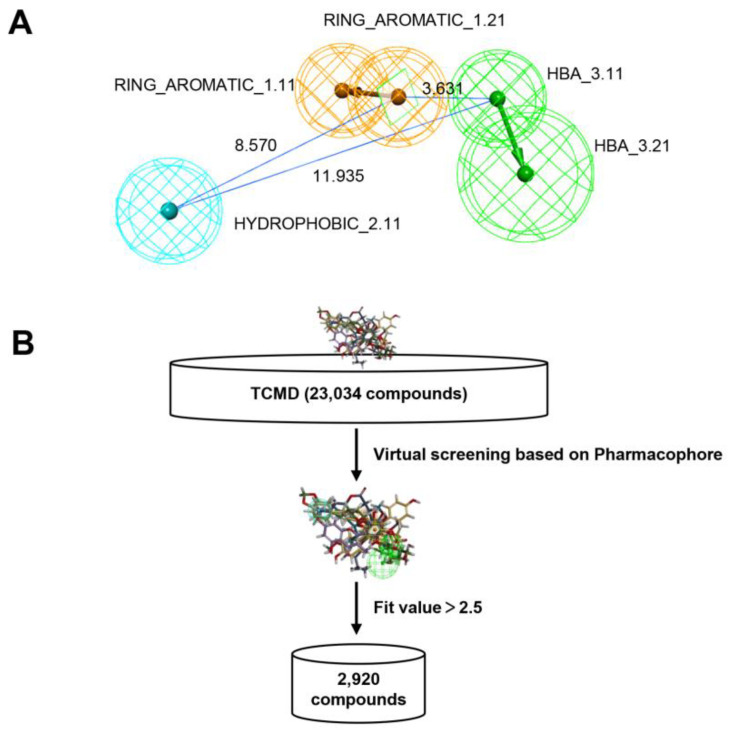
Virtual screening based on the pharmacophore modeling. (**A**) The structure of the best pharmacophore model (08), wherein green features represent hydrogen bond acceptor, blue features represent hydrophobic features and orange features represent ring aromatic, respectively. (**B**) The pharmacophore-based virtual screening process.

Specifically, pharmacophore model 08 consists of two hydrogen-bonded receptors, two aromatic ring centers, and one hydrophobic center (Figure 2A). Based on this model, the program of Search 3D Database in DS 2020 was used to screen potential eEF2K inhibitors from the traditional Chinese medicine database (TCMD) which contains 23,034 compounds. As shown in Figure 2B, a total of 2920 chemicals were obtained in this step of screen. The top 10 compounds in the fit value are shown in Table S1. To reduce the false-positive output of this step, a second step of screening is required.

### 2.3. Homology Modeling

**Figure 3:**
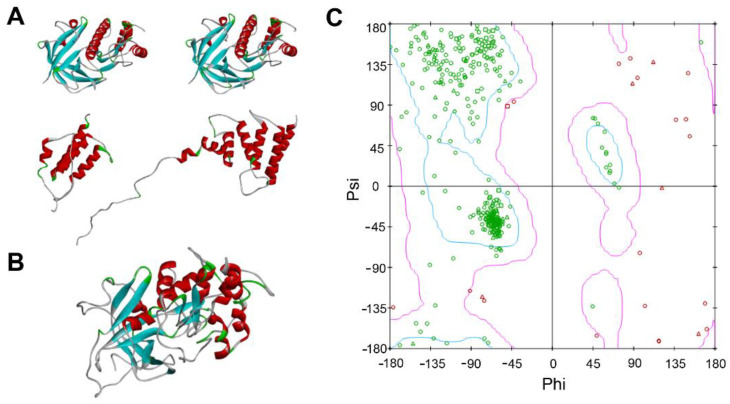
Homology modeling and evaluation. (**A**) The 3D structures of the template 6nx4_A, 5ks5_A, 3lkm_A, and 3pdt_A from the top left to the bottom right, respectively. (**B**) eEF2K 3D homology model (M0007). (**C**) Ramachandran map analysis of eEF2K 3D homology model.

eEF2K is a highly-conserved monomer protein and it consists of 725 amino acid residues, however, so far the intact 3D structure of eEF2K protein is not available in the PDB database. Thus, the Modeler module in DS 2020 was used to predict eEF2K protein structure based on its amino acid sequence. After searching the templates via the BLAST Search program, the protein crystals (i.e., 6nx4_A, 5ks5_A, 3lkm_A, and 3pdt_A) only with high homology to eEF2K_352–725_ were selected as templates for homology modeling (Figure 3A and Table S2). Interestingly, it was reported that eEF2K_562–725_ has a similar regulatory role with the full-length eEF2K on eEF2 [27], therefore it is feasible to use 3D structure of eEF2K_352–725_ to perform the molecular docking. Because of that, the 3D structure of eEF2K_352–725_ was constructed by using Build Homology Models in DS 2020, and in the end, a total of 10 protein models were generated (Supplementary Materials Figure S2).The lower the PDF Total Energy of the model, the better the reliability of the model. Thus, we analyzed the PDF Total Energy of each model, and found M0007 of the model, whose structure is shown in Figure 3B, had the lowest PDF Total Energy (Table 2), suggesting M0007 is the best model for eEF2K protein. Then, to further validate this 3D model of eEF2K protein, we depicted the distribution of Ramachandran map (Figure 3C), which is often used to examine the backbone conformation of each residue in a protein. From the map, we found that about 95% amino acid residues of the eEF2K protein were distributed in the best region indicated by blue circles. Therefore, the structure of M0007 is reliable and can be used for the follow-up study.

### 2.4. Virtual Screening Based on Molecular Docking

**Figure 4:**
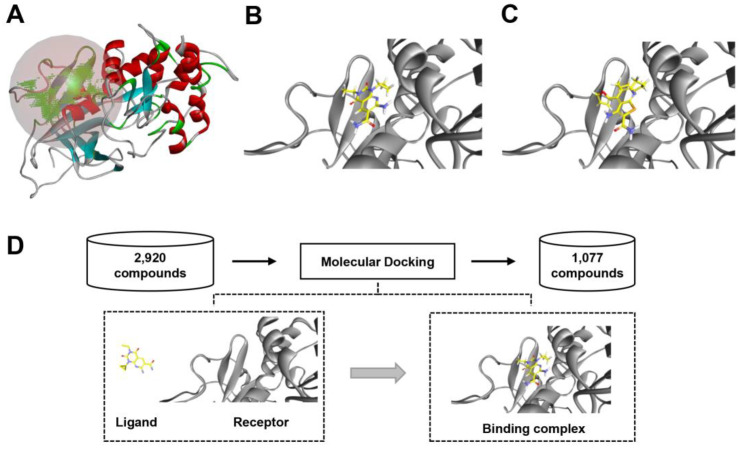
Molecular docking to screen chemicals interaction with eEF2K. (**A**) The defined inhibitor binding site to eEF2K protein, indicated by the circle. (**B**,**C**) The position of A484954 (**B**) and Compound **34** (**C**) in the binding pocket of eEF2K. (**D**) The work flow of molecular docking.

Next, to further screen eEF2K inhibitors from the previous output, the LibDock program of DS 2020 was used to select the chemicals which can be docked into the previously established 3D structure of eEF2K. Because the exact binding sites of inhibitors to eEF2K have not been uncovered, we defined the possible binding site in the protein cavity according to the structural characteristics of receptor (Figure 4A). To verify the validity of the predicted binding site, two well-known eEF2K inhibitors, A484954 and compound **34**, were used to simulate docking, and the results showed that both of them can bind to the predicted binding site (Figure 4B,C). Thus, this binding site is reliable and can be used for the following study. After docking screening, a total of 1077 compounds were obtained in the end (Figure 4D). Top 100 of the compounds are shown in Table S3. Of note, we found that most of the compounds in the list are not purchasable currently. To facilitate the follow-up experimental validation, the top 10 purchasable chemicals were picked according to their LibDock score from high to low, and then the list was re-ranked according to chemicals economic cost from low to high (Table 3). In the follow-up study, we mainly focused on these 10 chemicals.

### 2.5. Characterization of the Binding of Rhoifolin and Oleuropein to eEF2K

**Figure 5:**
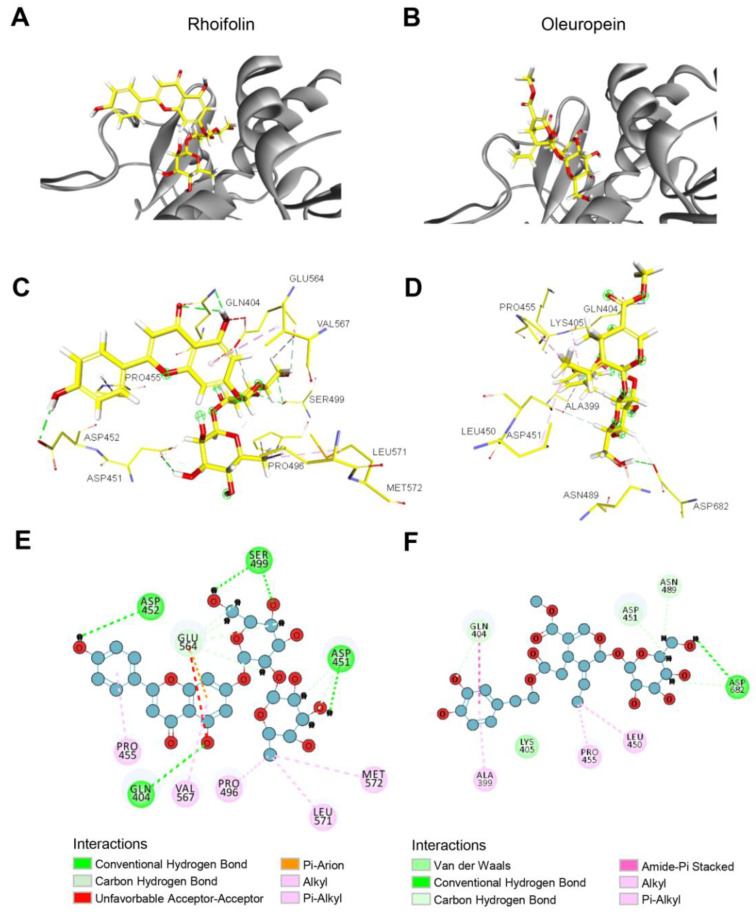
The predicted position of Rhoifolin and Oleuropein in the binding pocket of eEF2K. (**A**,**B**) The predicted binding mode of Rhoifolin (**A**) and Oleuropein (**B**) with eEF2K. (**C**,**D**) Ligand interactions of Rhoifolin (**C**) and Oleuropein (**D**) with eEF2K residues. (**E**,**F**) 2D interaction diagram of Rhoifolin (**E**) and Oleuropein (**F**) with eEF2K residues.

To validate the reliability of the molecular docking screen, the selected top 10 purchasable chemicals were docked with the assigned cavity of eEF2K. As shown in Figure 5A,B and Supplementary Figure S3, all 10 compounds can bind to the predicted binding site, suggesting our docking screen is successful. To more clearly exhibit the interaction of the hits with eEF2K, 3D images of the interaction between two examples (Rhoifolin and Oleuropein) with eEF2K were shown in Supplementary Figure S4. Given that the cost of a drug partially determines whether it can be applied at a large scale, two cheapest chemicals in the top 10, i.e., Rhoifolin and Oleuropein, were chosen to perform the subsequent analysis and in vitro experimental study.

Firstly, to closely observe the binding of selected chemicals to the eEF2K protein, we analyzed the non-bonding interaction (Figure 5C,D) and 2D receptor-ligand interaction (Figure 5E,F). The results indicated that Rhoifolin forms a hydrogen bond interaction with residue Gln404, Asp451, Asp452, and Ser499, and forms a hydrophobic interaction with Pro455, Pro496, Leu571, and Met572, respectively (Figure 5C,E). By contrast, Oleuropein forms a hydrogen bond interaction with residue Asp682, and forms a Van Der Waals force with residue Lys405 (Figure 5D,F). In addition, there was an amide-pi stacked interaction between Oleuropein and Gln404 residue (Figure 5F).

Additionally, we counted all the interactions between 1077 potential inhibitors with eEF2K residues, and found that the residues that form the most hydrogen bonds with the chemicals are Ser499, Pro401, and Pro455 (Supplementary Materials Figure S5A), and the residues that form the most hydrophobic interaction with the chemicals are Ser499, Ala399, and Pro401 (Supplementary Materials Figure S5B), and the residues that form the most charge interaction with the chemicals are Ser499 and Pro401 (Supplementary Materials Figure S5C), respectively. Overall, Ser499, Ala399, Pro455, and Pro401 were observed to have the highest counts of the favorable interactions (Supplementary Materials Figure S5D), suggesting these residues may play a key role in the interaction with the inhibitors of eEF2K and could be used as a reference for screening new inhibitors of eEF2K.

### 2.6. Evaluation of Effects of Rhoifolin and Oleuropein on eEF2K Activity

**Figure 6:**
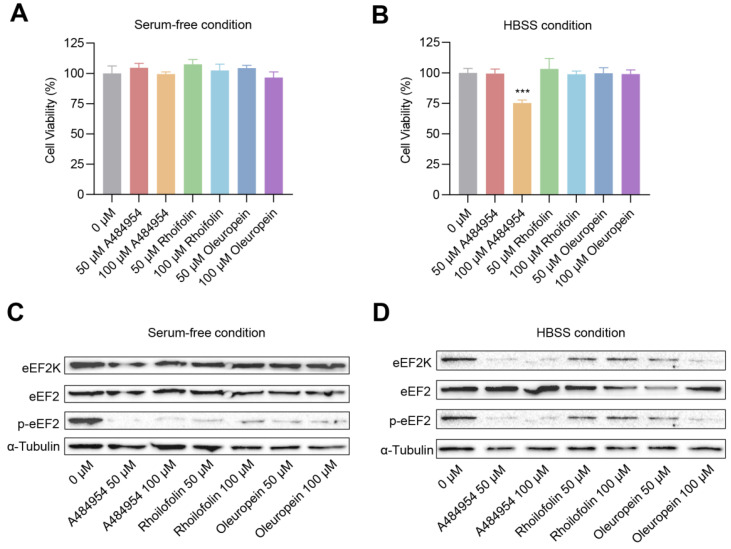
Effects of the selected compounds on cell viability and eEF2K activity. A484954 was used as a positive control. (**A**,**B**) CCK-8 assay of different concentrations of Rhoifolin and Oleuropein on Hela cells viability under serum-free condition (**A**) or HBSS condition (**B**), respectively (*n* = 4). (**C**,**D**) Western blotting analysis of treatments of different concentrations of Rhoifolin and Oleuropein on indicated protein levels under serum-free condition (**C**) or HBSS condition (**D**), respectively.

Next, to directly validate whether Rhoifolin and Oleuropein can inhibit eEF2K activity, Hela cells were used as an in vitro model, and the effects of Rhoifolin and Oleuropein on eEF2K activity were verified under two deprived nutrition conditions as previous reported [14]. A known eEF2K inhibitor, A484954, was adopted as a positive control. Firstly, to test the cytotoxicity of the chemicals, CCK8 (Cell Counting Kit-8) colorimetric assays were performed to detect cell viability after chemicals treatment. As shown in Figure 6A, in serum-free conditions, all the three chemicals showed undetectable cytotoxicity on Hela cells at 50 μM or 100 μM concentrations. Similar results were also observed in condition of Hanks’ Balanced Salt Solution (HBSS), except for 100 μM of A484954 treatment, which showed slight cytotoxicity on Hela cells (Figure 6B). Thus, these data indicate that 50 μM~100 μM of Rhoifolin or Oleuropein is a safe concentration to Hela cells.

Then, we detected the effects of Rhoifolin and Oleuropein treatments on eEF2K activity by western blotting. As shown in Figure 6C, under serum-free condition, both levels of A484954 did not affect the protein levels of eEF2K and its target eEF2, but dramatically reduced the phosphorylation levels of eEF2. This expected result suggests that our experimental system is reliable. Strikingly, a similar result was also observed in Rhoifolin and Oleuropein treatment, indicating Rhoifolin and Oleuropein are two new eEF2K inhibitors (Figure 6C). Subsequently, we verified the effects in HBSS conditions. The result showed that both Rhoifolin and Oleuropein addition significantly reduced the protein levels of phosphorylated eEF2, and relatively, the inhibitory effect of Oleuropein was more potent than Rhoifolin (Figure 6D). Of note, interestingly, the protein levels of eEF2K and eEF2 were also downregulated by treatment of A484954, Rhoifolin, or Oleuropein under HBSS condition (Figure 6D). Overall, these data demonstrate that Rhoifolin and Oleuropein indeed could inhibit eEF2K activity, suggesting Rhoifolin and Oleuropein are two new inhibitors of eEF2K.

### 2.7. Molecular Dynamics Simulation

**Figure 7:**
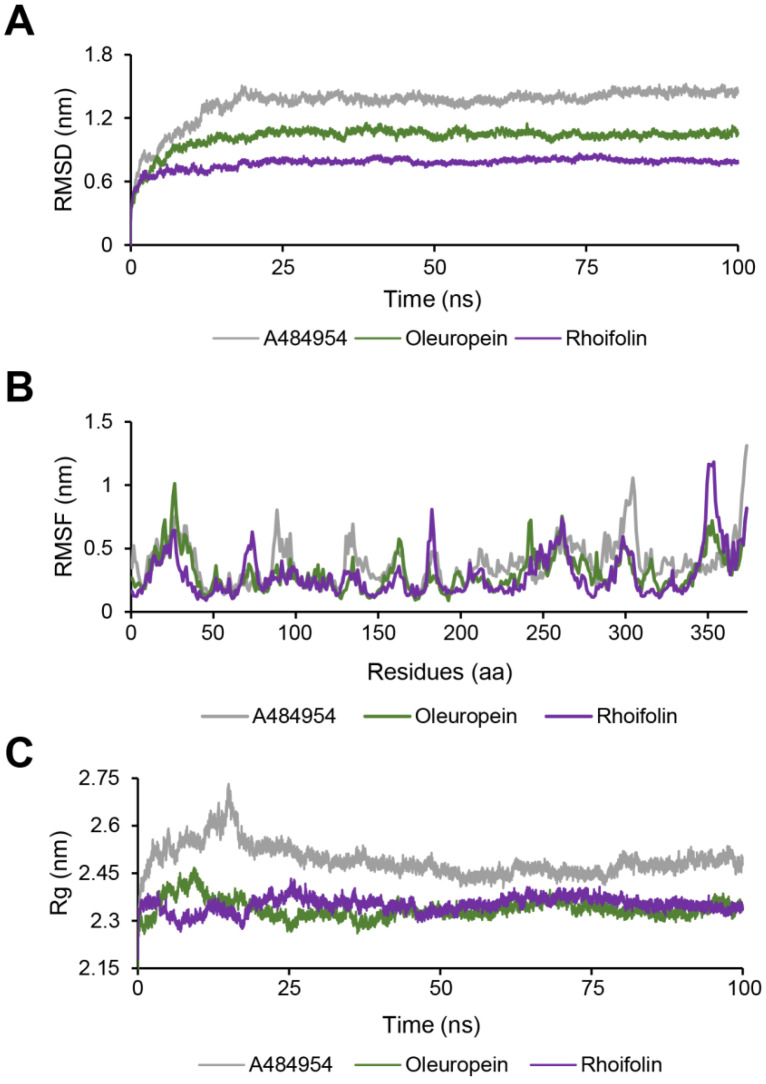
MD simulation analyses of eEF2K complexed to A484954, Oleuropein, and Rhoifolin. (**A**) RMSD values. (**B**) RMSF values. (**C**) Rg values. All analysis were performed with Gromacs.

Next, to evaluate the dynamic binding stability of Oleuropein and Rhoifolin in the complex of eEF2K, 100 ns MD simulations were employed, and A484954 was used as a reference ligand. First, we performed the RMSD measurement, which aims to calculate the average changes in atom displacement for evaluating the conformational shifts and biosystem stability. As shown in Figure 7A, the RMSD of A484954 gradually increased over the first 20 ns to about 1.3 nm, stabilizing to the end of operation, but fluctuations of about 1.2 to 1.4 nm were observed during most of the simulation. By contrast, the RMSD of Oleuropein and Rhoifolin were both lower than that of A484954. Between them, the better performer was Rhoifolin, which reached equilibrium around 10 ns and remained around 0.75 nm with minimal volatility. These observations suggest that Oleuropein and Rhoifolin have higher stability than A484954 in the complex with eEF2K. Then, RMSF analysis, which helps to explain the protein areas that fluctuate throughout the simulation, was conducted. The RMSF plot indicated that compared to A484954, Rhoifolin and Oleuropein exhibited relatively less fluctuations of the atoms in the complex (Figure 7B). Furthermore, we analyzed the Rg values of the protein-ligand complexes to examine changes in their densification throughout the simulation. The Rg plot showed that there was a large fluctuation with Rg value up to 2.53 nm within 25 ns in A484954-eEF2K complex and the average Rg value was 2.49 nm (Figure 7C). However, the average Rg value of Oleuropein-eEF2K complex was 2.34 nm and that of Rhofolin-eEF2K complex was 2.35 nm, which represent a narrower range of fluctuations compared to A484954 (Figure 7C). Collectively, MD simulations reflected that the Rhoifolin- or Oleuropein- eEF2K complex had the higher stability than the control, suggesting Rhoifolin and Oleuropein are promising to be potent inhibitors of eEF2K.

### 2.8. Pharmacokinetic and Toxicological Analyses

Finally, to explore the possibility of being drugs in the future, we predicted the pharmacokinetics and toxicity of Oleuropein and Rhoifolin by using online tools Swiss-ADME and eMolTox, respectively. As shown in Table 4, both chemicals had lower gastrointestinal absorption, suggesting it is necessary to develop a formulation in the future that allows their non-intestinal administration. Moreover, the two chemicals cannot cross the blood-brain barrier (Table 4), indicating they may have no effect on the central nervous system. However, both drugs can act as substrates of P-glycoprotein (P-gp) (Table 4), which may cause them to be pumped out of the cell [28], thus further chemical modifications are needed for future drug formation. Additionally, neither compound inhibits cytochromes (Table 4), suggesting they may not affect drug metabolism.

Next, the toxicological analysis showed that both compounds were not toxic to the central nervous system and were also not carcinogenic or genetically mutagenic (Table 5). However, in contrast to Oleuropein, Rhoifolin may have cardiotoxicity due to being a possible modulator of the platelet activating factor receptor which is associated with myocardial inflammation (Table 5) [29]. Collectively, these analyses indicate that Oleuropein and Rhoifolin may have lower toxicity, thus they can be considered as potential drugs.

## 3. Discussion

In recent years, along with the increased potential of eEF2K as a drug target in cancer, as well as in cardiovascular and neurodegenerative diseases, a growing attention has been paid to the development of eEF2K inhibitors [30]. However, to date no suitably potent inhibitors of eEF2K have been developed [13]. In this study, through combining in silico screening and in vitro biochemical analysis, we discovered two new inhibitors and numerous inhibitor candidates from the traditional Chinese medicine database. The findings here will provide a valuable resource for further identification of selective and potent eEF2K inhibitors.

Currently, computer-aided virtual screening technology is becoming prevalent in the new drug discovery. DS 2020 represents a powerful simulation tool as it allows a determination of compound binding sites, pharmacophore modeling and screening, drug design, and optimization [31]. Therefore, in this study we used this software to perform virtual screening. To increase the reliability of the screen, we integrated ligand-based pharmacophore modeling and molecular docking methods. Particularly, due to the lack of intact crystal structure of eEF2K in the PDB database, we constructed the 3D structure of the eEF2K protein through homology modeling (Figure 2A). While we selected the best model from the total 10 protein structure candidates for the follow-up study, we have to admit that there is still a gap between the predicted structures with the real structure. Thus, to facilitate eEF2K-targeting drug development, it is urgent to generate the refined crystal structure of eEF2K by traditional cryo-electron microscopy (cryo-EM) technology [32] or newly emerged artificial intelligence, such as AlphaFold [33] and RoseTTA-fold [34] in the future.

While great progress has been made in the virtual screening technology, it is still necessary to verify the validity. Among of the inhibitor hits, Rhoifolin and Oleuropein were selected to test the accuracy and reliability of the screen. They were selected following the criterion of the higher LibDock score and lower price in the rank. Specifically, Rhoifolin is a natural glycoside of apigenin, isolated from the green leaves of Rhus succedanea [35], while Oleuropein is a non-toxic secoiridoid derived from the olive tree [36]. To test the effects of the chemicals on eEF2K activity, we focused on the alteration of phosphorylation levels of eEF2, which is a primary downstream target of eEF2K and is often used as an indicator for eEF2K activity [5,18]. Of note, since eEF2K is inactive under adequate nutrition condition, thus two poor nutrition conditions, namely serum-free condition and HBSS condition, were adopted to test eEF2K activity just as previous reported [14]. To our surprise, both chemicals showed strong inhibitory effects on eEF2K activity as the phosphorylation levels of eEF2 is dramatically decreased (Figure 6C,D). However, we noticed a previous unreported phenomenon that, under HBSS conditions, the protein levels of eEF2K are also remarkably reduced after the treatment of these chemicals, including the positive control. Although we currently do not know the underlying mechanism, this finding indicates that the chemicals may exert functions via different manners depending on the specific conditions. Importantly, consistent with our finding, we noticed that both Rhoifolin and Oleuropein have been reported to have potent antiproliferative activity in several cancer cell lines [37,38], further suggesting that our screen strategy is reliable. On the other hand, our study uncovers a previous unknown mechanism that the anti-tumor properties of Rhoifolin and Oleuropein may be partially mediated by the inhibition of eEF2K activity. However, the selectivity of these two chemicals needs further investigation in the future.

Although in this study only Rhoifolin and Oleuropein on eEF2K activity were verified by in vitro studies, the remaining candidates, especially with high LibDock score in the rank, also have high potential to be eEF2K inhibitors. For example, Vitamin K2, which ranks in the top 10 purchasable candidates, has a high possibility to be a new eEF2K inhibitor because it has been reported to induce lung carcinoma cell apoptosis [39]. Thus, in the future, it would be worth conducting more biological studies to verify the candidate hits function, so as to identify selective and potent inhibitors of eEF2K or to seek inhibitors with unique structure which can be used as scaffolds for the optimization of eEF2K inhibitors. Additionally, while positive hits were obtained in this study, an improvement to the screen strategy could be applied in the future. The modified screen strategy is as follows: Firstly, virtual screening is performed on the whole TCMD by both molecular docking and pharmacophore modeling. Secondly, the intersection is obtained between the outcomes of the two screenings. Lastly, the compounds within the intersection can be selected as lead hits for the follow-up analysis. This new strategy can be tested in the future, which would be more effective in new drug discovery.

## 4. Materials and Methods

### 4.1. Construction of Pharmacophore Model

#### 4.1.1. Preparation of the Training Set Molecules

Based on the literature reports, the known eEF2K inhibitors were used as training sets and input into DS 2020 (Accelrys, San Diego, CA, USA). Then, to accurately distinguish the active from the inactive, two important parameters, i.e., Principal value and MaxOmitFix, were set as “2” and “0”, respectively. Principal value defines the level of molecular activity, and “2” means the molecule is a reference molecule and all chemical features in the molecule are considered when constructing the pharmacophore model. Moreover, MaxOmitFix defines the number of characteristic elements in each molecule that are allowed to not match the pharmacophore model, and “0” thereby represents that all the characteristic elements in the constructed pharmacophore model must match the compound.

#### 4.1.2. Common Feature Pharmacophore Generation and Evaluation

Pharmacophore was constructed by the Hiphop method based on common molecular features as described previously [40]. Briefly, the Feature Mapping tool was used to select possible pharmacophore characteristic elements. Then, Common Feature Pharmacophore Generation was employed to set Input Ligands as all small molecules in the training sets, and meanwhile set Conformation Generation as “Best”. In the end, 10 pharmacophore models were generated.

To select the best pharmacophore model for the follow-up study, the following steps were performed. Firstly, the test set, which includes both active and inactive compounds for eEF2K protein, was established for verifying the model. Then, the fit value between the pharmacophores and the chemicals in the test set was obtained by using the program of Ligand Profiler. Finally, pharmacophore models were evaluated by fit value-dependent indexes, such as hit rate of active compounds (HRA), identify effective index (IEI), and comprehensive appraisal index (CAI) [41]. Specifically, HRA represents the ability to identify active compounds from the test set, IEI means the ability of the model to identify active compounds from inactive compounds, and CAI is a comprehensive evaluation model of pharmacophore. These indexes are calculated by the following formulas, in which HA represents the number of active hit compounds from the test set, A represents the number of active compounds in the test set, Ht is the total number of hit compounds from the test set, and D represents the total number of compounds in the test set.

(1) $$\mathrm{HRA}=\left(\frac{\mathrm{HA}}{A}\right)\times 100\%$$

(2) $$\mathrm{IEI}=\frac{\left(\frac{\mathrm{HA}}{\mathrm{Ht}}\right)}{\frac{A}{D}}$$

(3) CAI=HRA×IEI

#### 4.1.3. Discovery of Lead Compounds

Traditional Chinese medicine database (TCMD, Version 2009), which contains 23,034 chemicals, was initially input into DS 2020 through Build 3D Database tool. Then, Search 3D Database program was used to screen the lead compounds in TCMD based on pharmacophore. Finally, the compounds with Fit value >2.5 were selected for follow-up analysis.

### 4.2. Homology Modeling

The eEF2K protein structure was predicted by method of homology modeling following previous study [42]. Briefly, the whole amino acid sequence of eEF2K was firstly retrieved from National Center for Biotechnology Information (NCBI) (https://www.ncbi.nlm.nih.gov/, accessed on 29 July 2022). Then, the Blast Search program of DS 2020 was adopted to search the templates in the Protein Data Bank (PDB) database (http://www.rcsb.org/pdb, accessed on 29 July 2022). The templates were selected mainly based on the homology and E-value, which represents the sequence similarity and template reliability, respectively. Generally, the higher the sequence homology and the lower the E-value, the better the templates. Therefore, the templates having a high sequence homology (>30) to eEF2K, but having a low E-value (<e-10), were chosen to load the structure through Load Structure and Alignment program of DS 2020. Subsequently, the 3D structure of eEF2K was built by using the Build Homology Model program in DS 2020 based on these template proteins. After that, the best 3D model of eEF2K protein was selected according to the indexes of PDF Total Energy and Ramachandran Plot, which are two often used parameters for evaluation the quality of 3D model

### 4.3. Virtual Screening Based on Molecular Docking

Since there is no referable inhibitor binding site for eEF2K protein, the possible interaction site was defined according to the receptor cavities (XYZ: −11.517721 1.429773 9.231896; Radius: 15.00000). Then, the 3D structure of eEF2K was prepared using the Prepare Protein program of DS 2020. After that, virtual screening was carried out using the LibDock module of DS 2020. LibDock is a rigid-based docking module. It calculates hotspots for the protein using a grid placed into the binding site and polar and a nonpolar probe. The hotspots are further used to align the ligands to form favorable interactions. The Smart Minimizer algorithm and CHARMM force field were performed for ligand minimization. After minimization, all ligand poses were ranked based on the ligands score [43]. Of note, since the LibDock score is essentially a measure of strength of binding affinity but not binding energy between the ligand substrate and receptor protein, it is a positive, rather than a negative, value. Finally, the chemicals, of which LibDock score is higher than that of the positive controls (A484954 and Compound **34**), were kept as candidates for eEF2K inhibitors.

### 4.4. Chemicals

Rhoifolin (CAS: 17306-46-6) was purchased from Yuanye Bio-Technology Corp (Shanghai, China), and Oleuropein (CAS: 32619-42-4) was ordered from Nakeli Bio-Technology Corp (Chengdu, China). They were both dissolved in DMSO at the stock concentration of 20 mM and stored at −20 °C.

### 4.5. Cell Culture

Hela cells were cultured in Dulbecco’s modified Eagle medium (Gibco, NY, USA) containing 10% fetal bovine serum (Gibco) and 1% (*v*/*v*) antibiotic in a humidified incubator at 5% CO_2_ at 37 °C. When treating the cells with 0 μM (DMSO), 50 μM or 100 μM A484954/Rhoifolin/Oleuropein, the culture medium was replaced with serum-free medium or Hanks’ balanced salt solution (HBSS). 24 h later, the cells were collected for CCK-8 assay or western blotting.

### 4.6. Cell Viability Assay

CCK-8 solution kit (Yeasen, Shanghai, China) was used to measure the cell viability. Hela cells were seeded in 100 μL of DMEM in a 96-well plate and placed in a 5% CO_2_ incubator at 37 °C. After a 24 h period, different concentrations of compounds were added into the plates and incubated for another 24 h in a humidified incubator (37 °C, 5% CO_2_). Then, the culture medium was removed and replaced with 100 μL of fresh medium containing 10% CCK-8 solution. After incubation for 2 h at 37 °C, the absorbance was read at a wavelength of 450 nm using a microplate reader (Tecan, Männedorf, Switzerland). The results were expressed as mean ± SD. Significance was estimated by one-way ANOVA. A probability of *p* < 0.05 was considered to be statistically significant. The statistical analysis and figures were prepared using GraphPad Prism 8.0.

### 4.7. Western Blotting

Hela cells were lysed with RIPA lysis buffer containing protease and phosphatase inhibitors. Protein concentrations were determined using the BCA protein quantitative assay kit (Beyotime Biotechnology, Shanghai, China). After boiling with loading buffer, 10~20 μg of prepared proteins were separated by electrophoresis on SDS-polyacrylamide gels and then transferred onto a polyvinylidene fluoride membrane (PVDF) for 120 min at 400 A. Then, the PVDF was blocked with TBST containing 5% skim milk powder for 1 h at room temperature. The membranes were incubated with the primary antibody overnight at 4 °C. The following primary antibodies and dilutions were used: anti-eEF2K (3692S, 1:1000), anti-eEF2 (2332S, 1:1000), anti-phospho-eEF2 (Thr56) (2331S, 1:1000) and anti-α-tubulin antibody (2148S, 1:1000). They were all ordered from Cell Signaling Technology, Danvers, MA, USA. Next, the membranes were incubated with the goat anti-rabbit IgG (111-035-003, Jackson, West Grove, PA, USA, 1:3000) for 1 h at room temperature. After adding ECL ultra-sensitive luminescent liquid (Solarbio, Beijing, China), the Image Lab (Bio-Rad) was used to detect chemiluminescence signals.

### 4.8. Molecular Dynamic Simulation

Molecular dynamic (MD) simulation was performed using with Gromacs [44].The simulation conditions were a full-atom CHARMM 36 force field, intermolecular transferable potential water model 3P (TIP3P), and the addition of Na^+^ or Clˉ to balance the system. All simulations maintain neutral ionization, performed in a three-oblique square. A total of 5000 minimization steps were performed using the steepest descent method and the particle mesh Ewald (PME) method. After minimization, two balancing steps and one production step were performed in succession, both using the Leap-Frog algorithm and the Berndsen coupling to control pressure and temperature. The first equilibrium simulation is a 100 ps equilibrium simulation in 300 K NVT, followed by a 100 ps equilibrium simulation in NPT at a 1.0 bar setup. The production simulations were 100 ns long and coordinates were saved every 10 ps. All trajectories were corrected for the marginal effects of cyclical conditions. Root mean square deviation (RMSD), Root mean square fluctuation (RMSF), Radius of gyration (Rg) between protein and ligand were calculated, all considering the protein backbone alone or in conjunction with ligands.

### 4.9. Pharmacokinetics and Toxicological Analyses

The online tools of Swiss-ADME [45] (http://www.swissadme.ch/, accessed on 29 July 2022) and eMolTox [46] (http://xundrug.cn/moltox, accessed on 29 July 2022) were employed to predict the pharmacokinetic and toxicological properties of the selected eEF2K inhibitor hits, respectively. For all analyses, the following SMILES codes were used. Oleuropein: COC(=O)C1=CO[C@@H](O[C@@H]2O[C@H](CO)[C@@H](O)[C@H](O)[C@H]2O)\C(=C\C)[C@@H]1CC(=O)OCCC1=CC(O)=C(O)C=C1; Rhoifolin: C[C@@H]1O[C@@H](O [C@@H]2[C@@H](O)[C@H](O)[C@@H](CO)O[C@H]2OC2=CC3=C(C(O)=C2)C(=O)C=C(O3)C2=CC=C(O)C=C2)[C@H](O)[C@H](O)[C@H]1O.

## 5. Conclusions

Overall, this study supplies an efficient strategy for identification of novel eEF2K inhibitors from traditional Chinese medicine, which will provide a useful reference for exploring inhibitors for other proteins. Meanwhile, the current study identifies two cheap inhibitors for eEF2K, i.e., Rhoifolin and Oleuropein. While the specificity and effectiveness of these two inhibitors need be evaluated in the future studies, the current findings have definitely given insights into eEF2K-targeting new drug development and anticancer therapy.

## Figures and Tables

**Table 1:** The test scores of the pharmacophore models.

Pharmacophore	Features	Rank	D	A	Ha	Ht	HRA	IEI	CAI
08	HHA	188.548	19	13	12	18	92.31%	0.974359	0.899408
02	RHA	206.975	19	13	9	14	69.23%	0.93956	0.650465
06	RHA	200.597	19	13	9	14	69.23%	0.93956	0.650465
01	RHA	209.374	19	13	8	13	61.54%	0.899408	0.553482
09	RHA	187.640	19	13	5	8	38.46%	0.913462	0.351331
03	RHA	206.677	19	13	2	3	15.38%	0.974359	0.149901
05	RHA	201.811	19	13	2	4	15.38%	0.730769	0.112426
07	RHA	191.351	19	13	2	2	15.38%	1.461538	0.224852
04	RHA	201.811	19	13	0	0	0.00%	0	0
10	RHA	185.059	19	13	0	0	0.00%	0	0

**Table 2:** The PDF energy score of the 3D models of eEF2K protein.

Name	PDF Total Energy	PDF Physical Energy	DOPE Score
M0007	18,709.2754	2533.81030749	−20,998.439453
M0003	19,247.8340	2847.16375490001	−18,696.406250
M0004	19,468.7598	2588.140841099	−18,796.500000
M0006	19,751.0020	2865.9443023	−21,234.820312
M0008	20,003.1426	2772.2228905791	−22,023.769531
M0005	20,003.2539	2956.111978	−20,604.794922
M0009	20,415.1406	2995.0741653	−21,289.044922
M0002	20,913.9961	3115.7153669	−19,221.875000
M0001	21,081.1328	3155.63869406	−19,377.949219
M0010	22,975.1074	3739.238885	−19,577.082031

**Table 3:** Top 10 purchasable chemicals obtained by molecular docking.

Rank	Name	Source Plant	LibDock Score	Price
1	Oleuropein	Fraxinus chinensis, Ligustrum lucidum, Fraxinus japonica, Ligustrum japonicum, Olea europaea.	152.589	1.80 $/mg
2	Rhoifolin	Anabasis aphylla	153.939	5.85 $/mg
3	Vitamin K2	Hippophae rhamnoides.	152.964	39.46 $/mg
4	Licuroside	Glycyrrhiza sp	148.674	63.25 $/mg
5	Chrysophanol-8-*O*-β-d-(6′-*O*-galloyl)-glucopyranoside	Rheum hotaoense.	152.437	74.96 $/mg
6	Calyxin H	Alpinia pinnanensis	160.676	90.36 $/mg
7	Sanggenon G	Morus mongolica, Morus alba.	149.677	134.93 $/mg
8	Cannabisin D	Hyoscyamus niger	154.383	154.33 $/mg
9	Bis-5,5-nortrachelogenin	Wikstroemia indica	166.105	426.95 $/mg
10	Fortunellin	Fortunella margarita, Fortunella crassifolia	160.992	619.72 $/mg

Note: The price of compounds was obtained from websites of Yuanye Bio-Technology Corp (Shanghai, China), Topscience Corp (Shanghai, China), Winherb Medical Technology Corp (Shanghai, China), BioBioPha Corp (Kunming, China), Desite Corp (Chengdu, China) and Nakeli Bio-Technology Corp (Chengdu, China), respectively.

**Table 4:** Pharmacokinetic analyses of Oleuropein and Rhoifolin.

Pharmacokinetic Analyses	Oleuropein	Rhoifolin
GI absorption	Low	Low
BBB permeant	No	No
P-gp substrate	Yes	Yes
CYP1A2 inhibitor	No	No
CYP2C19 inhibitor	No	No
CYP2C9 inhibitor	No	No
CYP2D6 inhibitor	No	No
CYP3A4 inhibitor	No	No

**Table 5:** Toxicological analyses of Oleuropein and Rhoifolin.

Toxicological Analyses	Oleuropein	Rhoifolin
Cardiotoxicity	Negative	Positive, modulator of platelet activating factor receptor
CNS Toxicity	Negative	Negative
Mutagenicity Genotoxicity	Negative	Negative
Carcinogenicity	Negative	Negative

## Data Availability

All data are given in the main manuscript and supplementary materials.

## Supplementary Materials

The following supporting information can be downloaded at: https://www.mdpi.com/article/10.3390/molecules27154886/s1, Figure S1: 10 pharmacophore models based on the common characteristics of molecules; Figure S2: 10 predicted eEF2K 3D homology models based on homology modeling; Figure S3: The positions of top 8 of 10 purchasable compounds in the binding pocket of eEF2K3D homology model; Figure S4: 3D images of the interaction between Oleuropein and Rhoifolin with eEF2K. Figure S5: Residue Interaction Histograms; Table S1: Top 10 chemicals with the highest fit value by searching 3D database; Table S2: The information of the protein templates; Table S3: Top 100 chemicals obtained from LibDock screening.

## Sample Availability

Not available.
