# Supplementary material for: Discovery of New Inhibitors of eEF2K from Traditional Chinese Medicine Based on In Silico Screening and In Vitro Experimental Validation
Source: Molecules. 2022 Jul 30;27(15):4886. doi: 10.3390/molecules27154886 (PMC9369671; doi:10.3390/molecules27154886)
Supplement: Supplementary file 1 [file molecules-27-04886-s001.zip › molecules-Supplementary Figures.pdf]

Figure S1

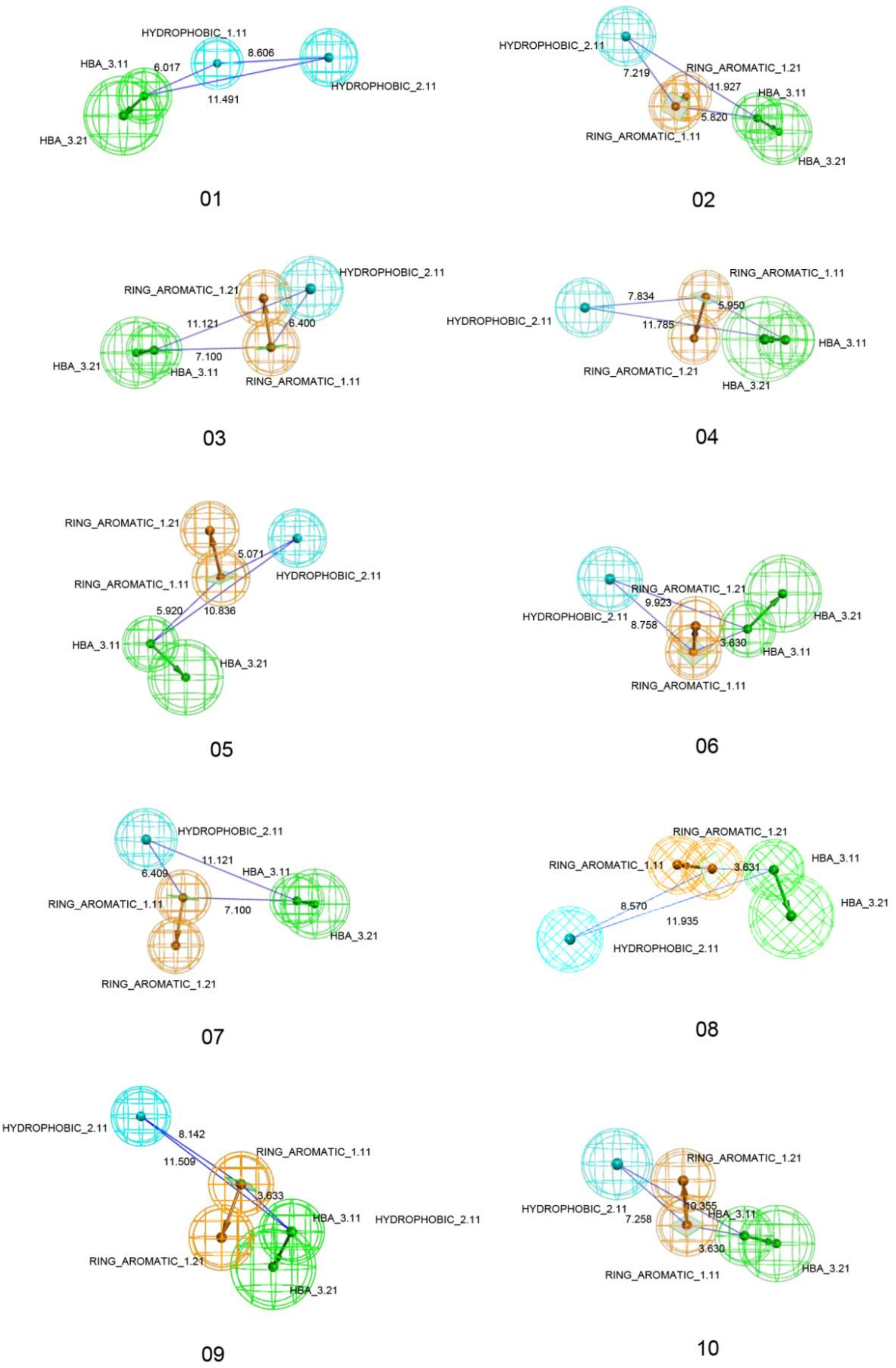

Figure S1. 10 pharmacophore models based on the common characteristics of molecules. Among them, green represents the hydrogen bond receptor, orange represents the aromatic ring center, and blue represents the hydrophobic center.

Figure S2

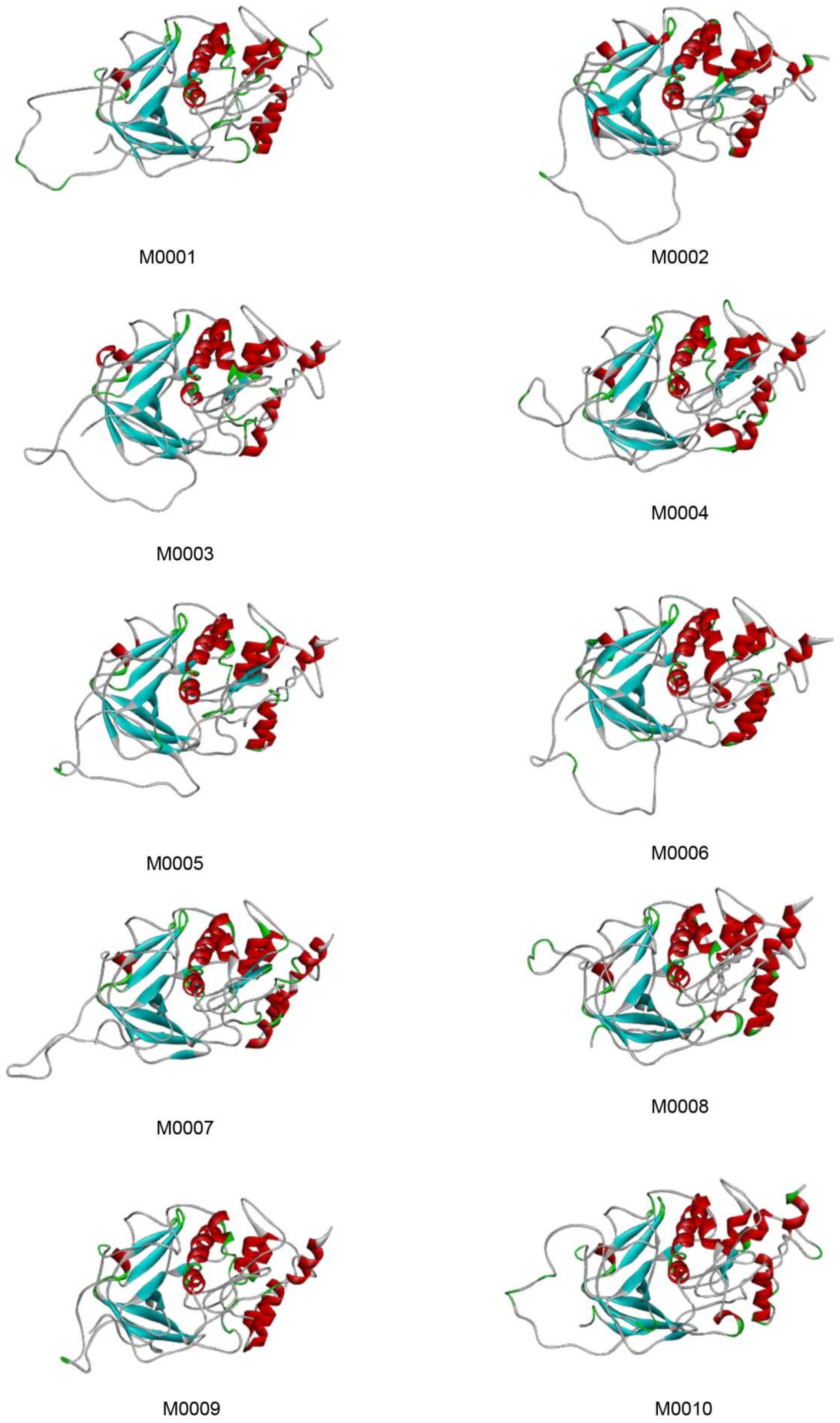

Figure S2. 10 predicted eEF2K crystal structures based on homology modeling.

Figure S3

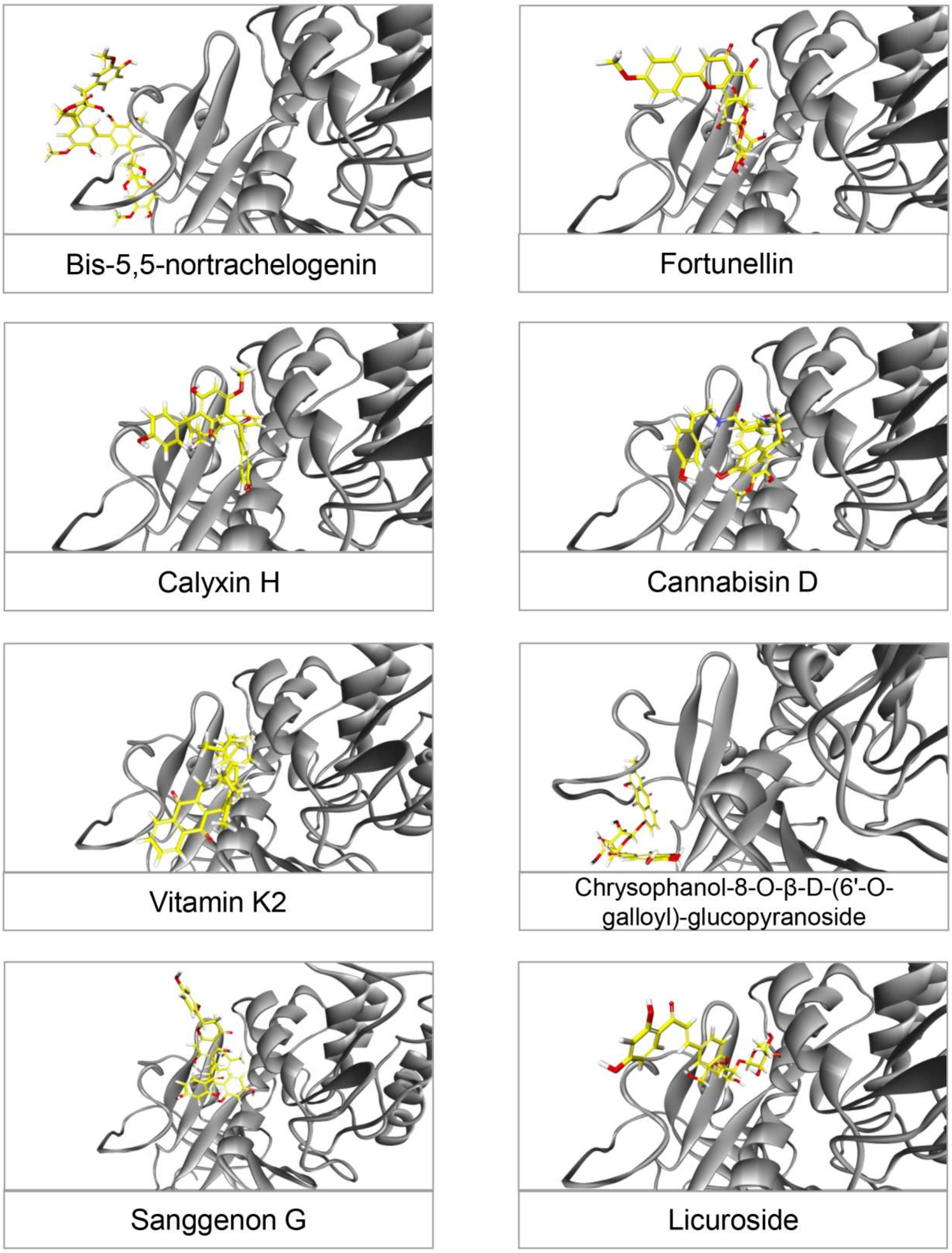

Figure S3. The positions of top 8 of 10 purchasable compounds in the binding pocket of eEF2K crystal structure.

Figure S4

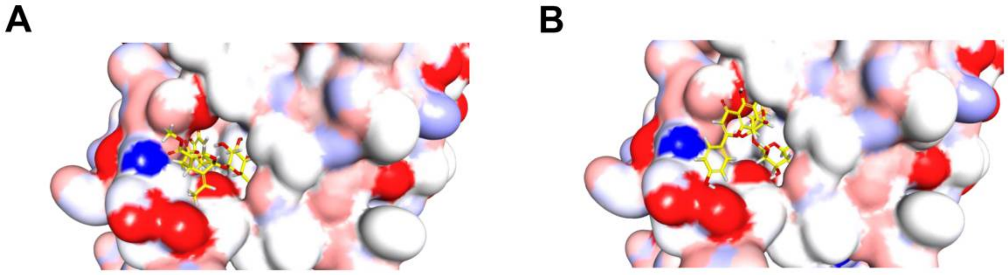

Figure S4. 3D images of the interaction between Oleuropein (A) and Rhoifolin (B) with eEF2K.

Figure S5

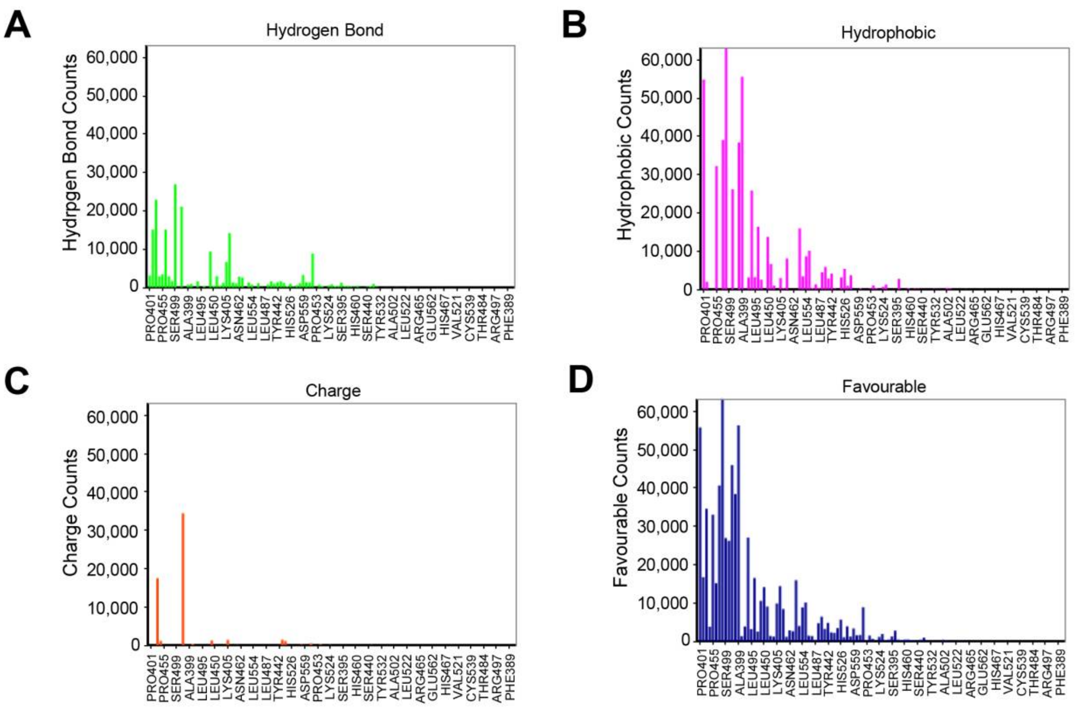

Figure S5. Residue Interaction Histograms. (A) Hydrogen bond interaction with residues histogram. (B) Hydrophobic interaction with residues histogram. (C) Charge interaction with residues histogram. (D) Favorable interaction with residues histogram.
