# Supplementary material for: Discovery of New Inhibitors of eEF2K from Traditional Chinese Medicine Based on In Silico Screening and In Vitro Experimental Validation
Source: Molecules. 2022 Jul 30;27(15):4886. doi: 10.3390/molecules27154886 (PMC9369671; doi:10.3390/molecules27154886)
Supplement: Supplementary file 1 [file molecules-27-04886-s001.zip › molecules-Supplemetary Tables.pdf]

Table S1. Top 10 chemicals obtained by searching 3D database

Rank	Name	Source plant	Fit value
1	Curtisian J	Paxillus curtisii	2.95072
2	Dichotomoside B	Stellaria dichotoma	2.94267
3	Quercetin-3-methyl-7-methyl ether-4'-sulfate	Ipomoea regnellii	2.93948
4	Styraxlignolide B	Styrax japonica .	2.9365
5	(2S,3S,1'S,2'R)-and(2S,3S,1'R,2'R)-2,3-Dihydro-5-(1',2'-dihydroxypropyl)-2-(4-hydroxy-3-methoxyphenyl)-7-methoxy-3-methylbenzofuran	Aristolochia pubescens	2.93541
6	Hericenone F	Hericium erinaceus	2.93007
7	4''',5,5'',7''-Tetrahydroxy-3''',4',7-trimethoxy-3,6''-biflavone	Aristolochia ridicula	2.92876
8	Tinosposide B	Tinospora sinensis	2.92715
9	Triisofuhalol octaacetate	Chorda filum	2.9215
10	Manassantin A	Saururus chinensis , Houttuynia cordata, Saururus sp	2.91953

Table S2. The information of the protein templates

Title	Accession	Sequence length	Bit score	Identity	E-value	Organism
Eukaryotic elongation factor 2 kinase	6nx4_A	167	256.914	80	1.39E-81	Homo sapiens
Myosin heavy chain kinase A	3lkm_A	247	163.696	36	1.02E-45	Dictyostelium discoideum
Myosin heavy chain kinase A	3pdt_A	251	162.925	36	1.97E-45	Dictyostelium discoideum
Eukaryotic elongation factor 2 kinase	5ks5_A	103	154.451	80	2.91E-44	Homo sapiens

Table S3. Top 100 eEF2K inhibitor hits obtained by LibDock screening

Rank	Name	Source plant	LibDock Score
1	Umbilicaxanthoside B	Umbilicaria proboscidea	171.438
2	Revandchinone 1	Rheum emodi	169.069
3	Hyacinthin	Hyacinthus orientalis, Vitis vinifera	169.040
4	Bis-5, 5-nortrachelogenin	Wikstroemia indica	166.105
5	Scabroside	Gentiana scabra	165.151
6	Hymenoside B	Hymenophyllum barbatum	163.587
7	Woorenoside V	Coptis japonica	163.333
8	Fortunellin	Fortunella margarita, Fortunella crassifolia	160.992
9	Calyxin H	Alpinia pinnanensis	160.676
10	Alnuside C	Alnus japonica	160.113
11	-Catechin-3-O--D-gluco -pyranoside	Inga umbellifera	159.311
12	-3, 5-Dihydroxy-1- -7- heptane 5-O--D-glucopyranoside	Tacca chantrieri	158.687
13	Revandchinone 4	Rheum emodi	158.683
14	Blepharocalyxin C	Alpinia blepharocalyx	158.503
15	-Syringoylglycerol 9-O- --D-glucopyranoside	Hyssopus officinalis	157.202
16	Magnolignan I	Magnolia officinalis	156.208
17	2', 7-Dihydroxy-4'-methoxy-8-prenylflavan 2', 7-di-O--D-glucopyranoside	Morus alba , Morus alba	156.054
18	Kompasinol A	Maackia amurensis, Caragana tibetica	155.960
19	Cannabisin D	Hyoscyamus niger	154.383
20	Rhoifolin	Ligustrum robustum, Lamiophlomis rotata , Poncirus trifoliata, Poncirus trifoliata, Toxicodendron succedaneum , Trachelospermum jasminoides, Rhus sylvestris, Citrus aurantium	153.939
21	Odontoside	Odontites serotina	153.803
22	7-O- secologanolic acid	Lonicera japonica	153.227
23	Vitamin K2	Hippophae rhamnoides	152.964
24	Murramarin A	Murraya exotica	152.936
25	Oleoyl neocryptotanshinone	Salvia miltiorrhiza	152.634
26	Oleuropein	Fraxinus chinensis, Ligustrum lucidum, Fraxinus japonica,	152.589

		Ligustrum japonicum, Olea europaea	
27	Chrysophanol-8-O--D- -glucopyranoside	Rheum hotaoense	152.437
28	GarcinianoneB	Garcinia multiflora	151.613
29	Kuwanon P	Morus macroura	151.383
30	-3, 5, 4'-Trihydroxystilbene 3-O--D-glucopyranoside	Eskemukerjea megacarpum	151.353
31	3, 7-Dihydroxy-2, 5-diundecylnaphthoquinone	Aegiceras corniculatum	150.935
32	Muscanone	Commiphora wightii	150.518
33	Sanggenon G	Morus mongolica , Morus alba	149.677
34	-3, 5-Dihydroxy-1, 7-bis heptane 3-O--D-glucopyranoside	Tacca chantrieri	149.583
35	1, 2-Di-O-E-sinapoyl--glucopyranose	Brassica napus	149.544
36	6'-O--D-Galactopyranosylsyringopicroside	Syringa amurensis	148.996
37	Licuroside	Glycyrrhiza sp	148.674
38	Hordatine B	Hordeum vulgare	148.105
39	Seguinoside F	Glycosmis pentaphylla	148.033
40	3, 5-Dihydroxy-stilbene-3-O-neohesperidoside	Dryopteris sublaeta	147.885
41	Neohesperidin	Poncirus trifoliata, Citrus limon, Citrus unshiu, Citrus aurantium	147.873
42	Quercetin-7-O- --glucopyranosyl- --rhamnopyranoside-3-O--glucopyranoside	Aconitum napellus ssp neomontanum	147.664
43	Naringin	Poncirus trifoliata, Poncirus trifoliata, Poncirus trifoliata, Drynaria fortunei, Dryopteris crassirhizoma, Citrus limon, Citrus limon, Citrus par adisi, Flemingia strobilifera, Origanum vulgare, Citrus grandis, Citrus aurantium, Citrus aurantium, Citrus decumana, Citrus unshiu , Adiant um sp	147.539
44	-2- -6, 7, 8-trihydroxy-5, 6, 7, 8-tetrahydro-5-chromone	Aquilaria agallocha	147.427
45	Mulberrofuran W	Morus mongolica	147.418
46	Lappaol B	Arctium lappa	147.252
47	Cochinchinone A	Cratoxylum cochinchinense	146.839
48	Alnuside B	Alnus japonica	146.741

49	6'', 7''-Dihydro-5', 5'''-dicapsaicin	Capsicum annuum	146.721
50	Ixerochinoside	Ixeris chinensis	146.680
51	Kukoamine A	Lycium chinense	146.606
52	3'-O-trans-Cinnamoyl-astilbin	Andira inermis	146.567
53	7-O-Methylaloeresin A	Aloe marlothii	146.472
54	3'-Prenyl-4'-methoxy-isoflavone-7-O'--D-glycopyranoside	Sopubia delphinifolia	146.377
55	10-Hydroxy-4-O-methyl-2, 11-diundecylgomphilactone	Aegiceras corniculatum	146.273
56	Hymenoside I	Hymenophyllum barbatum	146.213
57	Yemuoside YM1	Stauntonia chinensis	146.021
58	Hexaspermone C	Ochna macrocalyx, Ouratea hexasperma	146.006
59	Artocommunol CB	Artocarpus incisa	145.809
60	Salvianolic Acid B	Salvia miltiorrhiza	145.726
61	Urobilin	Homo sapiens	145.462
62	-3, 5, 3', 4'-Tetrahydroxystilbene 3-O--D-glucopyranoside	Eskemukerjea megacarpum	145.247
63	6''--D-Xylose-genistin	Glycine max	144.838
64	Procyanidin B5	Garcinia mangostana, Cinnamomum bejolghota , Pinus radiata, Davallia mariesii, Theobroma cacao, Campylotropis hirtella, Aesculus hippocastanum, Cinnamomum cassia , Dioscorea cirrhosa , Pinus taeda	144.626
65	Procyanidin B7	Rheum sp, Salix caprea, Cinnamomum cassia , Pinus taeda, Vaccinium vitis-idae a, Cinnamomum camphora	144.626
66	Diospyrosonaphthoside	Diospyros angustifolia	144.487
67	Ruixianglangdusu B	Stellera chamaejasme	144.466
68	Ruixianglangdusu A	Stellera chamaejasme	144.466
69	Silybin	Silybum marianum	144.431
70	Silybin B	Silybum marianum	144.431
71	AH21	Aquilaria agallocha	144.388
72	Procyanidin B5 3'-O-gallate	Vitis amurensis	144.233
73	Procyanidin B2-3''-O--D-allopyranoside	Pronephrium simplex	144.232
74	Saccatoside	Gmelina philippensis , Verbascum saccatum	144.225

75	Kushenol B	Sophora flavescens	144.147
76	Tenuiphenone C	Polygala tenuifolia	143.991
77	Ligustrosidic acid	Ligustrum lucidum	143.897
78	Protosappanin E1	Caesalpinia sappan	143.892
79	Protosappanin E2	Caesalpinia sappan	143.892
80	2-n-Pentacosyl-5, 7-dihydroxy-6, 8-dimethyl chromone	Sophora flavescens	143.735
81	Magnesium lithospermate	Cordia spinescens	143.675
82	-2- -6, 7, 8-trihydroxy-5, 6, 7, 8-tetrahydro-5- -chromone	Aquilaria agallocha	143.569
83	4-Methoxyphenyl 1-O--D- -apiofuranosyl- --D-glucopyranoside	Tabebuia impetiginosa	143.247
84	Odoratrin	Gnidia lamprantha, Daphne odora	143.239
85	Alpinnanin A	Alpinia pinnanensis	143.052
86	Alpinnanin B	Alpinia pinnanensis	143.052
87	2-Methoxy-5- -phenyl-1-O- --D-glucopyranoside	Baseonema acuminatum	143.022
88	Calyxin L	Alpinia blepharocalyx	142.964
89	Tribulusamide B	Tribulus terrestris	142.940
90	Dimeric 4, 2', 4'-trihydroxydihydrochalcone	Agapanthus africanus	142.779
91	-ent-18- -Coumaroyloxy-8 -labden-15-oic acid	Nuxia sphaerocephala	142.774
92	Brachystamide C	Piper brachystachyum	142.771
93	Trifochalcanoloside I	Trifolium alexandrinum	142.745
94	Hymenoside E	Hymenophyllum barbatum	142.696
95	Chrysophanol 8-O--D- glucopyranoside	Rheum qinlingense	142.689
96	Sigmoidin A	Erythrina abyssinica , Erythrina sigmoidea	142.667
97	Lappaol E	Arctium lappa	142.511
98	Violyedoenamide	Viola yedoensis	142.473
99	Epicalyxin I	Alpinia blepharocalyx	142.395
100	Hydroxygerambullol	Glycosmis chlorosperma	142.192

Note: The chemicals labeled in red, *i.e.*, Rhoifolin and Oleuropein, were selected for verifying the reliability of the screen.
